# Advances in the treatment of intracranial blood blister-like aneurysms: a comprehensive review from surgical approaches to endovascular therapy

**DOI:** 10.3389/fneur.2026.1952908

**Published:** 2026-10-13

**Authors:** Ganghua Feng, Yuanbiao Lei, Yi Zhang, Qiuli Li, Fuyong Ou, Le Zhang, Xiaoxi Yao

**Affiliations:** 1 Department of Neurology, The First People's Hospital of Chenzhou, Chenzhou, Hunan, China; 2 Department of Neurology, The First Clinical College of Xiangnan University, Chenzhou, Hunan, China; 3 Department of Neurology, The First Affiliated Hospital & Clinical Neuroscience Institute of Jinan University, Guangzhou, Guangdong, China

**Keywords:** bypass, clipping, endovascular therapy, flow-diverter, intracranial blood blister-like aneurysm, surgical management

## Abstract

Intracranial blood blister-like aneurysms (BBAs) constitute a rare but clinically challenging subset of cerebrovascular lesions, distinguished by their fragile, thin-walled morphology and a tendency to arise at non-branching sites of intracranial arteries, most frequently in the supraclinoid segment of the internal carotid artery (ICA). Managing BBAs is particularly difficult due to their high propensity for rupture, rapid progression, and the challenges they pose in intraoperative identification and repair. Traditional surgical approaches, including direct clipping, wrapping, and bypass with trapping, have been employed; however, these techniques are often associated with high complication rates and technical limitations given the delicate structure of BBAs. In recent years, advances in endovascular therapy, such as stent-assisted coiling (SAC) and flow-diverter (FD) placement, have expanded the therapeutic options, offering less invasive alternatives with encouraging outcomes. Nevertheless, the optimal management strategy remains controversial, as both surgical and endovascular approaches carry distinct indications, technical challenges, and risks of recurrence or procedural complications. This narrative review systematically explores the epidemiology and pathophysiology of BBAs, delineates key diagnostic features, and critically examines both reconstructive and deconstructive treatment modalities within a stepwise, two-pathway (endovascular and surgical) algorithm, highlighting reported efficacy, complication profiles, and recommended treatment selection for distinct aneurysm subtypes. By contrasting the strengths and limitations of each approach, this article aims to provide clinicians with evidence-based guidance to support individualized management strategies for patients with BBAs.

## 1 Introduction

Blood blister-like aneurysms (BBAs) constitute a distinct and highly challenging subset of intracranial aneurysms, distinguished by their unique epidemiological and anatomical characteristics (Figure 1). Unlike the more common saccular aneurysms, BBAs typically arise at non-branching sites of cerebral arteries, with a pronounced predilection for the supraclinoid segment of the internal carotid artery (ICA) (1, 2). Epidemiological studies indicate that BBAs represent a small fraction of all intracranial aneurysms, yet they are disproportionately associated with severe clinical outcomes, including high rates of rupture, morbidity, and mortality (3, 4). Their insidious nature, often presenting as acute subarachnoid hemorrhage (SAH), underscores their clinical significance and the urgent need for prompt diagnosis and effective management (5). Recent advances in imaging techniques, such as high-resolution vessel wall imaging (HR-VWI) and three-dimensional (3D) magnetic resonance imaging, have enhanced the detection of these elusive lesions, particularly in cases where conventional angiography fails to identify a causative abnormality (6, 7). Despite these technological improvements, the diagnosis and management of BBAs remain complex due to their subtle radiological features and aggressive clinical behavior.

**Figure 1:**
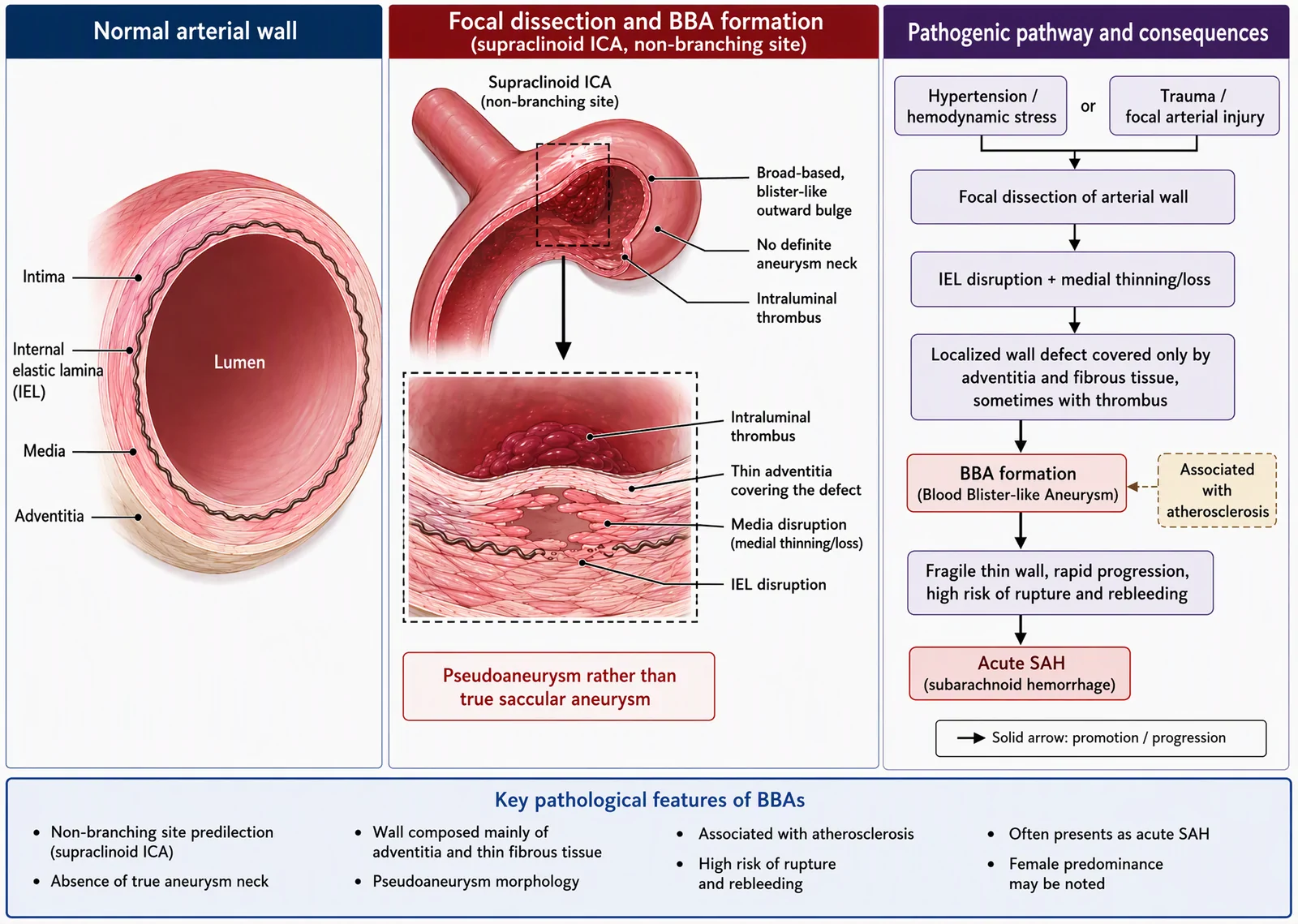
Pathophysiology of blood blister-like aneurysms (BBAs). **(A)** Normal arterial wall structure comprising the intima, internal elastic lamina (IEL), media, and adventitia. **(B)** Focal dissection and BBA formation characterized by disruption of the IEL and media, a thin residual adventitia, and intraluminal thrombus. **(C)** Pathogenic pathway from hypertension/trauma to endothelial injury, IEL disruption, and BBA formation. Key pathological features of BBAs include a predilection for non-branching sites (supraclinoid ICA), absence of a true neck, a wall composed only of adventitia and thin fibrous tissue, high rupture and rebleeding risk, pseudoaneurysm morphology, association with atherosclerosis, female predominance, and frequent presentation as acute subarachnoid hemorrhage (SAH). IEL, internal elastic lamina; ICA, internal carotid artery; SAH, subarachnoid hemorrhage.

The pathological characteristics of BBAs further complicate their clinical management. Unlike typical saccular aneurysms, BBAs are distinguished by their extremely thin, fragile walls and the absence of a well-defined aneurysmal neck (5, 8). Histopathological and molecular studies indicate that BBAs often represent pseudoaneurysms arising from focal dissections of the arterial wall, with a marked tendency for rapid growth, rebleeding, and recurrence (9, 10). The wall structure typically consists only of adventitia and a thin layer of fibrous tissue, lacking the normal three-layered architecture, which renders these lesions highly susceptible to rupture even with minimal manipulation (11). Recent single-cell transcriptomic analyses suggest a pathogenic link between preexisting intracranial atherosclerosis and BBA formation, highlighting the roles of inflammatory processes and mural cell dysfunction (9). The high risk of intraoperative rupture, coupled with dynamic morphological changes, underlies the poor clinical outcomes often associated with BBAs, including severe neurological deficits and mortality (11, 12). This pathological complexity underscores the need for a nuanced and carefully tailored approach to both diagnosis and intervention.

From a therapeutic standpoint, BBA management strategies can be broadly categorized into two conceptual frameworks: reconstructive techniques, which aim to preserve parent artery patency while excluding the aneurysm (e.g., direct clipping, wrapping-clipping, stent-assisted coiling, flow-diverter placement), and deconstructive techniques, which sacrifice the parent artery segment harboring the aneurysm, typically with concomitant bypass to maintain cerebral perfusion (e.g., trapping with high-flow extracranial-intracranial bypass). The selection between these approaches depends on aneurysm morphology, location, patient comorbidities, and institutional expertise (1, 3).

Historically, the management of BBAs has evolved in response to the considerable challenges posed by their pathology and clinical presentation. Traditional microsurgical approaches, such as direct clipping and wrapping, have been associated with high rates of intraoperative rupture, incomplete aneurysm exclusion, and poor neurological outcomes (11, 12). Although clip-reinforced wrapping and bypass procedures have offered some improvements, their technical complexity and variable efficacy have limited widespread adoption (13, 14). The introduction of endovascular therapies, including stent-assisted coiling, flow-diverters (FDs), and covered stents, has significantly expanded the therapeutic options for BBAs (2, 4, 15). These minimally invasive approaches aim to reconstruct the parent vessel and facilitate aneurysm healing while reducing the risks of rebleeding and ischemic complications. Nonetheless, each modality carries distinct technical challenges, risk profiles, and long-term outcomes, rendering the selection of the optimal treatment strategy a complex, patient-specific decision (3).

In recent years, the emergence of hybrid treatment strategies and novel devices has further reshaped the management of BBAs. The integration of microsurgical and endovascular techniques in hybrid operating suites, alongside the development of advanced flow-diverting and covered stents, has facilitated more tailored and effective interventions for this high-risk patient population (1, 15). Systematic reviews and meta-analyses have begun to elucidate the relative efficacy and safety of these approaches; however, consensus on the optimal management of BBAs remains elusive (2–4). The ongoing evolution of diagnostic and therapeutic modalities underscores the need for continuous appraisal of clinical evidence to guide decision-making and improve patient outcomes.

The purpose of this narrative review is to provide a comprehensive, up-to-date synthesis of advancements in the treatment of intracranial BBAs, encompassing both traditional surgical approaches and the latest endovascular techniques. We aim to systematically examine the indications, efficacy, and limitations of each method, drawing upon current literature and clinical evidence (2, 3, 14). By delineating the strengths and weaknesses of available therapies within reconstructive and deconstructive frameworks, this review seeks to inform clinical decision-making and identify areas for future research in the management of this challenging cerebrovascular entity.

## 2 Methods

This narrative review was conducted to summarize current evidence on the diagnosis and treatment of intracranial blood blister-like aneurysms (BBAs). A comprehensive literature search was performed using PubMed/MEDLINE, Embase, and Web of Science databases. The search strategy employed combinations of the following terms: “blood blister-like aneurysm,” “blister aneurysm,” “dorsal internal carotid artery aneurysm,” “intracranial pseudoaneurysm,” “endovascular treatment,” “flow-diverter,” “stent-assisted coiling,” “surgical clipping,” “wrapping,” “bypass,” and “trapping.” No language restrictions were applied; however, only articles published in English or with available English translations were included. The search covered publications from database inception through December 2024.

Inclusion criteria encompassed original research articles, systematic reviews, meta-analyses, clinical series, and case reports describing the diagnosis, treatment, or outcomes of intracranial BBAs. Exclusion criteria included studies focusing exclusively on non-intracranial aneurysms, non-BBA aneurysm types, or those lacking sufficient clinical or technical detail. Bibliographies of retrieved articles were manually screened to identify additional relevant studies.

This study is a narrative review, not a systematic review or meta-analysis; accordingly, no formal systematic-review protocol (such as PRISMA), meta-analytic framework, or risk-of-bias assessment was applied, and no statistical pooling was performed. The evidence is described qualitatively, with emphasis on the hierarchy of study designs (from randomized controlled trials to case series and reports) where applicable. To strengthen the synthesis, we incorporated quantitative outcome data (e.g., occlusion rates, complication rates, and functional outcomes) from the cited systematic reviews and meta-analyses where such data were available. Treatment modalities are discussed within a reconstructive-versus-deconstructive conceptual framework and organized into a stepwise, two-pathway (endovascular and surgical) algorithm to facilitate clinical decision-making. Given the rarity of BBAs and the predominance of retrospective studies and case series in the literature, the evidence base remains limited, and recommendations should be interpreted accordingly.

### 2.1 Clinical and imaging characteristics of intracranial BBAs

#### 2.1.1 Epidemiology and pathogenesis

BBAs are rare, fragile vascular lesions that predominantly arise in the supraclinoid segment of the ICA, characterized by their small size, thin walls, and lack of a well-defined neck. Epidemiologically, BBAs are more frequently observed in middle-aged women, with multiple studies reporting a notable female predominance. For example, a systematic review examining FD monotherapy for BBAs reported a mean patient age of approximately 50 years, with 73.4% of cases occurring in females, highlighting this demographic tendency (2). Similarly, a clinical series utilizing the Willis covered stent (WCS) for BBA treatment included predominantly female patients (15 out of 21), with a mean age of 51.6 years, further corroborating that women with hypertension are disproportionately affected (15).

Hypertension is frequently identified as a significant risk factor for BBA formation, often coexisting with these aneurysms and potentially contributing to their pathogenesis through chronic hemodynamic stress on the arterial wall (3, 16). However, the precise causal relationship between hypertension and BBA development remains incompletely understood, and further prospective studies are warranted to clarify this association.

Pathologically, BBAs are considered pseudoaneurysms rather than true saccular aneurysms. Their formation is closely linked to focal arterial wall injury, particularly involving the internal elastic lamina and tunica media, resulting in a localized defect covered only by a thin adventitial layer or thrombus. This structural vulnerability underlies their high propensity for rupture and rebleeding. Recent advances in molecular pathology, including single-cell RNA sequencing, have illuminated the inflammatory and atherosclerotic milieu associated with BBA development. These studies demonstrate a predominance of macrophages and a relative paucity of mural cells, alongside upregulation of genes related to arterial mineralization and atherosclerosis, suggesting that preexisting intracranial atherosclerosis may predispose the parent artery to BBA formation (9). Additionally, imaging and surgical reports indicate that BBAs often originate from acute focal dissections of the vessel wall, supporting the hypothesis that local arterial injury and subsequent pseudoaneurysm formation are central to their pathogenesis (10). Advanced imaging modalities, such as HR-VWI MRI, have proven valuable in detecting these subtle wall abnormalities, emphasizing the importance of vascular wall pathology in both diagnosis and mechanistic understanding of BBAs (5, 6). Collectively, these findings highlight the unique epidemiological profile and complex pathobiology of BBAs, with a strong predilection for middle-aged women with hypertension and a pathogenesis rooted in focal arterial wall injury, pseudoaneurysm formation, and underlying atherosclerotic changes.

#### 2.1.2 Key points in imaging diagnosis

The imaging diagnosis of BBAs poses considerable challenges due to their small size, fragile structure, and atypical morphology. Conventional imaging modalities, such as digital subtraction angiography (DSA), computed tomography angiography (CTA), and magnetic resonance angiography (MRA), are routinely employed for aneurysm detection but have notable limitations in the context of BBAs. Although DSA is considered the gold standard, it may fail to detect BBAs, particularly in the acute phase, owing to their subtle, non-saccular appearance. Certain angiographic features, however, can enhance detection, including irregularities in the parent artery diameter, delayed contrast outflow, and stagnation of contrast along the aneurysm wall. These characteristics help distinguish BBAs from typical saccular aneurysms, which generally present with a well-defined neck and dome (16).

Recent advances in HR-VWI, particularly 3D magnetic resonance techniques, have markedly enhanced the sensitivity for detecting BBAs. Techniques such as 3D HR-VWI MRI and contrast-enhanced VWI (CE-VWI) provide superior contrast between the aneurysm wall and surrounding tissues, enabling improved visualization of subtle vascular abnormalities. These methods can detect wall enhancement, which often reflects active pathology such as dissection or rupture, and have proven effective in identifying BBAs that are occult on conventional imaging. HR-VWI has demonstrated the ability to differentiate BBAs from other vascular lesions and to influence clinical management by accurately localizing the bleeding source in cases of SAH with negative initial angiography (5–7).

Intraoperative indocyanine green (ICG) fluorescence angiography serves as a valuable adjunct during microsurgical aneurysm repair. We emphasize that ICG angiography is not used for the primary diagnosis of BBAs, which relies on DSA, CTA, MRA, and particularly HR-VWI; rather, ICG provides real-time visualization of blood flow dynamics and vessel patency during microsurgical procedures, aiding in the intraoperative confirmation of aneurysm exclusion and the assessment of parent artery and branch integrity. This is particularly valuable for BBAs, where the absence of a well-defined neck complicates treatment. The role of ICG in confirming successful aneurysm occlusion and preserving critical vessels during aneurysm microsurgery is supported by its established intraoperative use as an adjunct to decision-making (16, 17).

### 2.2 Surgical treatment

Surgical management of BBAs encompasses both reconstructive and deconstructive strategies. Reconstructive approaches aim to preserve parent artery patency while reinforcing or excluding the aneurysm (direct clipping, wrapping-clipping), whereas deconstructive approaches involve parent artery sacrifice with concurrent revascularization to prevent ischemic complications (trapping with bypass) (Figure 2).

**Figure 2:**
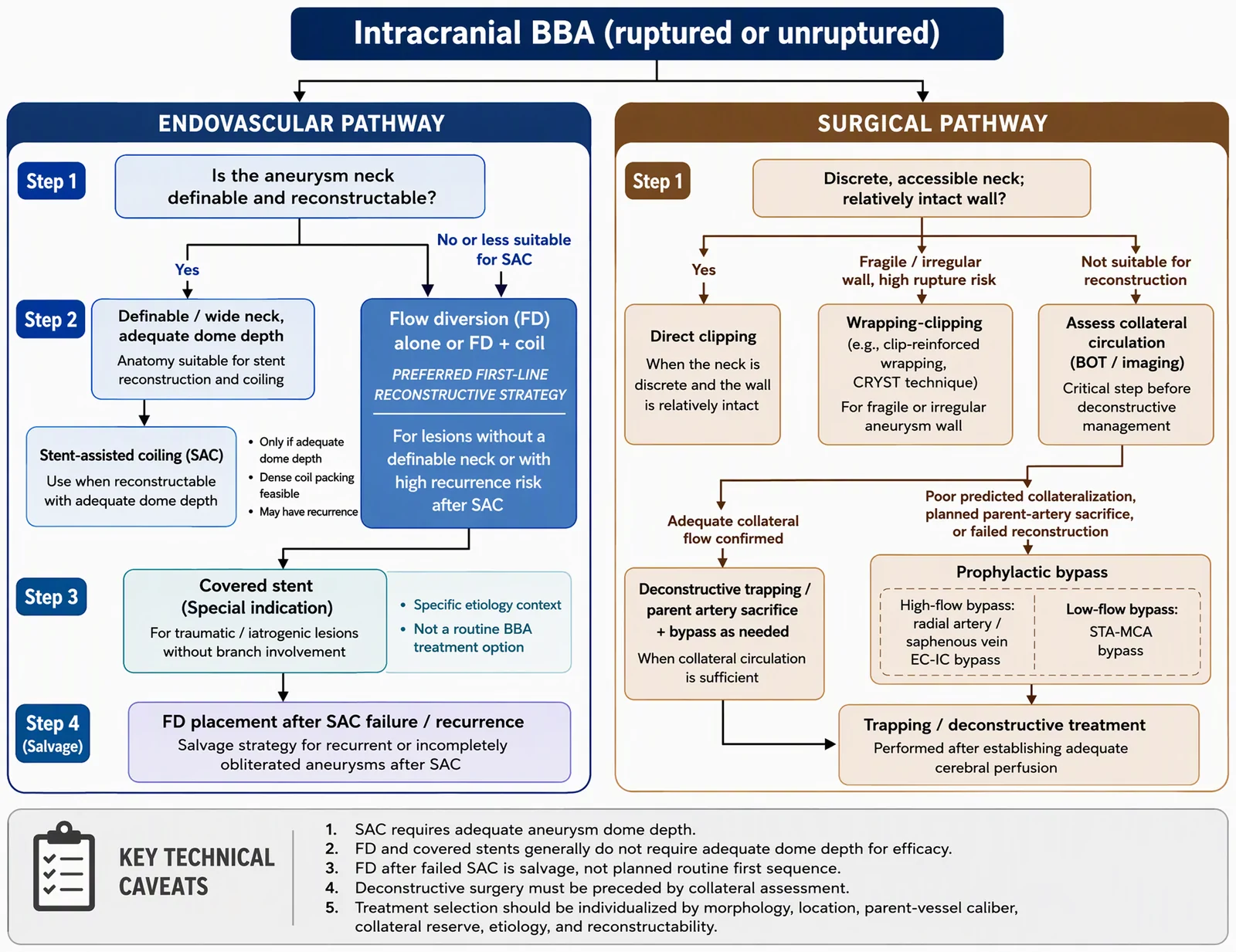
Proposed stepwise treatment algorithm for intracranial BBAs, divided into endovascular and surgical treatment pathways. The endovascular pathway proceeds from stent-assisted coiling (SAC; for lesions with a definable, wide neck and adequate dome depth) to flow-diverter (FD) alone or FD + coil (preferred first-line combination for lesions without a definable neck), covered stents (for traumatic/iatrogenic lesions without branch involvement), and salvage FD after SAC failure. The surgical pathway proceeds from direct clipping (discrete, accessible neck with relatively intact wall) to wrapping–clipping (fragile/irregular wall; e.g., CRYST technique), and, for lesions unsuitable for reconstruction, to collateral assessment (balloon occlusion test [BOT]/imaging) followed by deconstructive trapping with high-flow (radial artery or saphenous vein EC-IC bypass) or low-flow (STA–MCA bypass) revascularization; prophylactic bypass is indicated for patients with poor predicted collateralization, planned parent-artery sacrifice, or failed reconstruction. Key technical caveats: SAC requires adequate aneurysm dome depth for dense coil packing, whereas FD and covered stents do not require dome-depth considerations; tiny (≤2 mm) lesions and small parent-vessel caliber impose additional technical challenges. Traumatic/iatrogenic lesions and basilar perforator microaneurysms share a blister-like morphology but differ in pathogenesis from true ICA BBAs and should be managed according to their specific etiology. BOT, balloon occlusion test; CRYST, clip-reinforced wrapping using the Y-shaped temporalis fascia; EC-IC, extracranial-intracranial; FD, flow-diverter; ICA, internal carotid artery; MCA, middle cerebral artery; SAC, stent-assisted coiling; STA, superficial temporal artery.

#### 2.2.1 Direct clipping and wrapping-clipping techniques (reconstructive)

Direct clipping of BBAs presents considerable technical challenges due to the extremely thin and fragile aneurysmal wall, which is highly susceptible to tearing during manipulation. This inherent fragility increases the risk of intraoperative rupture and clip slippage, resulting in high rates of procedural failure and postoperative rebleeding. Clinical reports have shown that even after initially successful direct clipping, aneurysm regrowth or clip displacement may occur, sometimes necessitating additional interventions such as high-flow bypass to achieve definitive aneurysm exclusion (11).

To mitigate these risks, wrapping-clipping techniques have been developed, employing materials such as Bemsheet or autologous fascia to reinforce the aneurysm wall before or during clip application. This approach enhances aneurysmal structural integrity, reduces the likelihood of intraoperative rupture, and diminishes the risk of postoperative rebleeding. For example, the clip-reinforced wrapping using the Y-shaped temporalis fascia (CRYST) technique has demonstrated favorable outcomes in BBA patients, with no severe complications, neurological deficits, or aneurysm recurrence observed during follow-up. Postoperative angiography confirmed parent artery patency with minimal or reversible stenosis, supporting the safety and efficacy of this method (13). These findings indicate that wrapping-clipping techniques, by strengthening the aneurysm wall and stabilizing the clip, offer a valuable reconstructive alternative to direct clipping, particularly for BBAs with irregular morphology and high rupture risk.

#### 2.2.2 Bypass and trapping techniques (deconstructive)

High-flow extracranial-intracranial (EC-IC) bypass, often using radial artery grafts or saphenous vein grafts, combined with aneurysm trapping (segmental occlusion), has emerged as a key deconstructive therapeutic strategy for managing complex BBAs, particularly those located on the supraclinoid ICA. This approach is primarily indicated for fragile, broad-based aneurysms lacking a defined neck, which are highly prone to re-rupture and are often unsuitable for direct clipping or endovascular coiling due to their morphology and wall fragility (1).

The choice of graft material and bypass flow capacity depends on individual anatomical and hemodynamic considerations. High-flow bypass (radial artery or saphenous vein grafts) is preferred when collateral circulation is insufficient, when a large dependent territory (e.g., the entire MCA distribution) is at risk, or when planned sacrifice of the ICA is required; radial artery grafts are commonly employed due to their favorable caliber match with the MCA and excellent long-term patency, whereas saphenous vein grafts are an important alternative when the radial artery is unavailable, of insufficient caliber, or when higher flow rates are required (18). By contrast, low-flow bypass (superficial temporal artery [STA]–MCA anastomosis) may be appropriate when the hemodynamic demand is lower and the donor/recipient anatomy is favorable, such as in selected distal lesions or when a robust collateral network already exists. A recent systematic review of EC-IC bypass for complex ICA aneurysms demonstrated that both radial artery and saphenous vein grafts can achieve favorable outcomes, with graft patency rates exceeding 90% at follow-up (18). The selection of graft material and bypass type should be individualized based on preoperative assessment of vessel caliber, flow requirements, collateral reserve, and donor site availability.

The procedure typically involves creating the selected EC-IC bypass, such as an anastomosis between the external carotid artery (or STA for low-flow) and the MCA using the chosen graft, to secure adequate cerebral perfusion prior to trapping or occluding the aneurysmal segment. This staged strategy maintains continuous blood flow to dependent brain territories, thereby minimizing the risk of ischemic complications. The protective role of bypass-trapping in preventing intraoperative catastrophic hemorrhage is well established. By providing a robust alternative route for cerebral blood flow prior to aneurysm segment occlusion, this approach significantly reduces the risk of sudden, uncontrollable bleeding during manipulation of the fragile aneurysm wall (1, 11). Prophylactic bypass is indicated for patients in whom poor collateralization is predicted on preoperative imaging (e.g., by balloon occlusion test or imaging-based collateral assessment), for those planned for parent artery sacrifice or aggressive trapping, and for those in whom reconstruction has failed or is unfeasible. Clinical series have reported favorable neurological outcomes following this combined strategy, with most patients achieving good functional recovery (modified Rankin Scale scores of 0–2 at follow-up) and no evidence of aneurysm recurrence or bypass graft failure on imaging (1). Although the procedure is technically demanding, requiring meticulous planning and execution by experienced neurovascular teams, the use of high-flow bypass substantially mitigates the risk of ischemic injury, making it a valuable deconstructive option for otherwise untreatable BBAs.

#### 2.2.3 Intraoperative complications and prognosis

In the treatment of intracranial BBAs, intraoperative complications, such as aneurysm rupture, cerebral vasospasm, and cerebral infarction, remain major concerns that directly influence patient prognosis. Intraoperative rupture is a critical event, often resulting in catastrophic hemorrhage and increased morbidity and mortality. While both surgical and endovascular approaches carry this risk, advances in endovascular techniques, including balloon-assisted coiling, stent-assisted coiling, and flow diversion, have enhanced technical control, enabling more precise device deployment and aneurysm exclusion. Nevertheless, the risk persists, particularly in fragile, thin-walled BBAs, which are highly susceptible to rupture during manipulation.

Cerebral vasospasm represents another significant complication, arising either secondary to SAH or from intraoperative vessel irritation. Balloon angioplasty has been used effectively to treat symptomatic vasospasm, improving neurological outcomes in a substantial proportion of patients (19). Cerebral infarction, often resulting from thromboembolic events or inadvertent vessel occlusion during aneurysm treatment, is also a notable concern, especially when aneurysms are located near critical perforating arteries or within the posterior circulation, where ischemic sequelae are more likely (20). Preoperative imaging evaluation, including assessment of collateral circulation and vessel dominance, such as the anterior communicating artery, plays a crucial role in predicting postoperative ischemic risk. Patients with anatomical variants or poor collateralization identified on imaging are at higher risk for ischemic complications following parent artery occlusion or aggressive aneurysm trapping and may therefore benefit from planned bypass procedures or alternative strategies (20, 21). Overall, while modern endovascular and microsurgical techniques have reduced the incidence of severe complications, meticulous preoperative planning and intraoperative vigilance remain essential for optimizing outcomes.

### 2.3 Advances in endovascular therapy

Endovascular treatment of BBAs represents a predominantly reconstructive approach, aiming to preserve parent artery patency while promoting aneurysm thrombosis and healing.

#### 2.3.1 Stent-assisted coil embolization (SAC)

SAC has emerged as a pivotal reconstructive technique in the management of BBAs, particularly for aneurysms with wide necks or unfavorable dome-to-neck ratios that preclude simple coiling. The procedure involves deploying a stent across the aneurysm neck to serve as a scaffold, preventing coil herniation into the parent vessel and enabling dense coil packing within the aneurysm sac. Critical technical considerations include the selection of appropriate stent types, such as low-profile visualized intraluminal support (LVIS), Neuroform Atlas, Enterprise 2, and LEO stents, each offering distinct properties in terms of flexibility, metal coverage, and vessel wall apposition (22–26).

In the management of acutely ruptured BBAs, SAC has demonstrated favorable efficacy but is associated with several limitations. Clinical studies and case series report high rates of immediate and short-term aneurysm occlusion, with complete obliteration exceeding 85–90% in selected cohorts (22, 27–29). SAC effectively reduces hemodynamic stress on the fragile aneurysm dome, thereby minimizing the risk of early rebleeding, a major concern during the acute phase (28, 30). However, the unique pathology of BBAs, characterized by a thin, fragile wall and poorly defined neck, presents technical challenges, including coil instability, incomplete neck coverage, and a predisposition to aneurysm recurrence or regrowth. An important technical consideration is that SAC requires an adequate aneurysm dome depth to permit dense coil packing and to prevent coil herniation or instability; shallow or very shallow BBAs with limited dome depth are therefore poorly suited to SAC alone. Residual neck or incomplete occlusion remains a concern, with recurrence rates reported between 20–30% in pooled analyses, necessitating close angiographic follow-up and, in some cases, retreatment (29, 31).

Given the high recurrence rate and technical challenges of SAC alone in BBAs, the combination of a flow-diverter with coil embolization (FD + coil) is increasingly preferred as a first-line reconstructive strategy for ruptured BBAs, rather than a staged plan of SAC followed by delayed FD after recurrence. This combined approach leverages the immediate protection against rebleeding afforded by coil packing in the acute phase while the FD provides definitive hemodynamic remodeling and durable parent-artery reconstruction (32, 33). Quantitative data support this strategy: in a pooled analysis of ICA BBAs treated with FDs, the complete occlusion rate was higher with FD + coil (~98%) than with FD alone (~87%), with correspondingly favorable clinical outcomes (~93.1% vs. ~ 81.9%) (33). A staged strategy of SAC followed by delayed FD placement should therefore be reserved for selected cases of recurrence after initial SAC, and is not recommended as a planned first-line sequence, because it exposes patients to multiple procedures and cumulative antiplatelet-related risk (33, 34).

#### 2.3.2 Flow-diverter (FD) devices

The advent and evolution of FD devices, including the Pipeline embolization device (PED), Flow Redirection Endoluminal Device (FRED), and Surpass Evolve, have substantially transformed the reconstructive therapeutic landscape for BBAs and other complex aneurysms. In a systematic review of FD monotherapy for BBAs, most cases involved the ICA, with complete occlusion rates reaching 88.1% and favorable clinical outcomes in 82.2% of patients, highlighting robust aneurysm protection and parent artery patency (2). Real-world experience further supports FD use, even in acutely ruptured aneurysms; a single-center study reported a complete embolization rate of 94.7% and favorable outcomes in 90.4% of patients when FDs were combined with adjunctive coil embolization, despite a small risk of ischemic complications (35). Newer-generation devices such as FRED and Surpass Evolve are designed to optimize deployment, reduce thrombogenicity, and improve navigability, offering advantages for anatomically challenging lesions.

The application of FDs across various anatomical locations, including the ICA, vertebral artery, and basilar artery, demonstrates variable efficacy and safety, largely influenced by vessel size, branch anatomy, and local hemodynamics. In the ICA, particularly for BBAs, FDs have become the preferred reconstructive treatment modality due to their ability to reconstruct the parent artery and promote aneurysm healing (2, 36). Importantly, for FD or covered-stent strategies, adequate aneurysm dome depth is generally not required, because aneurysm occlusion relies on flow remodeling (FD) or physical exclusion (covered stent) rather than on intrasaccular packing; this contrasts with SAC, for which dome depth is a relevant technical consideration. In the posterior circulation, including the vertebral and basilar arteries, FD use is more nuanced. Case reports and small series have demonstrated successful aneurysm occlusion and parent artery reconstruction with devices such as Pipeline and Silk Vista Baby, even in challenging scenarios like dissecting aneurysms of the posterior inferior cerebellar artery (PICA) and vertebrobasilar fenestration aneurysms (37, 38). However, these locations carry higher risks of ischemic complications due to critical perforating arteries and the need for meticulous antiplatelet management. Staged treatment strategies, such as initial coiling followed by delayed FD placement, have also been explored for ruptured aneurysms in delicate locations, demonstrating high rates of aneurysm occlusion with minimal morbidity or mortality (Figure 3) (39).

**Figure 3:**
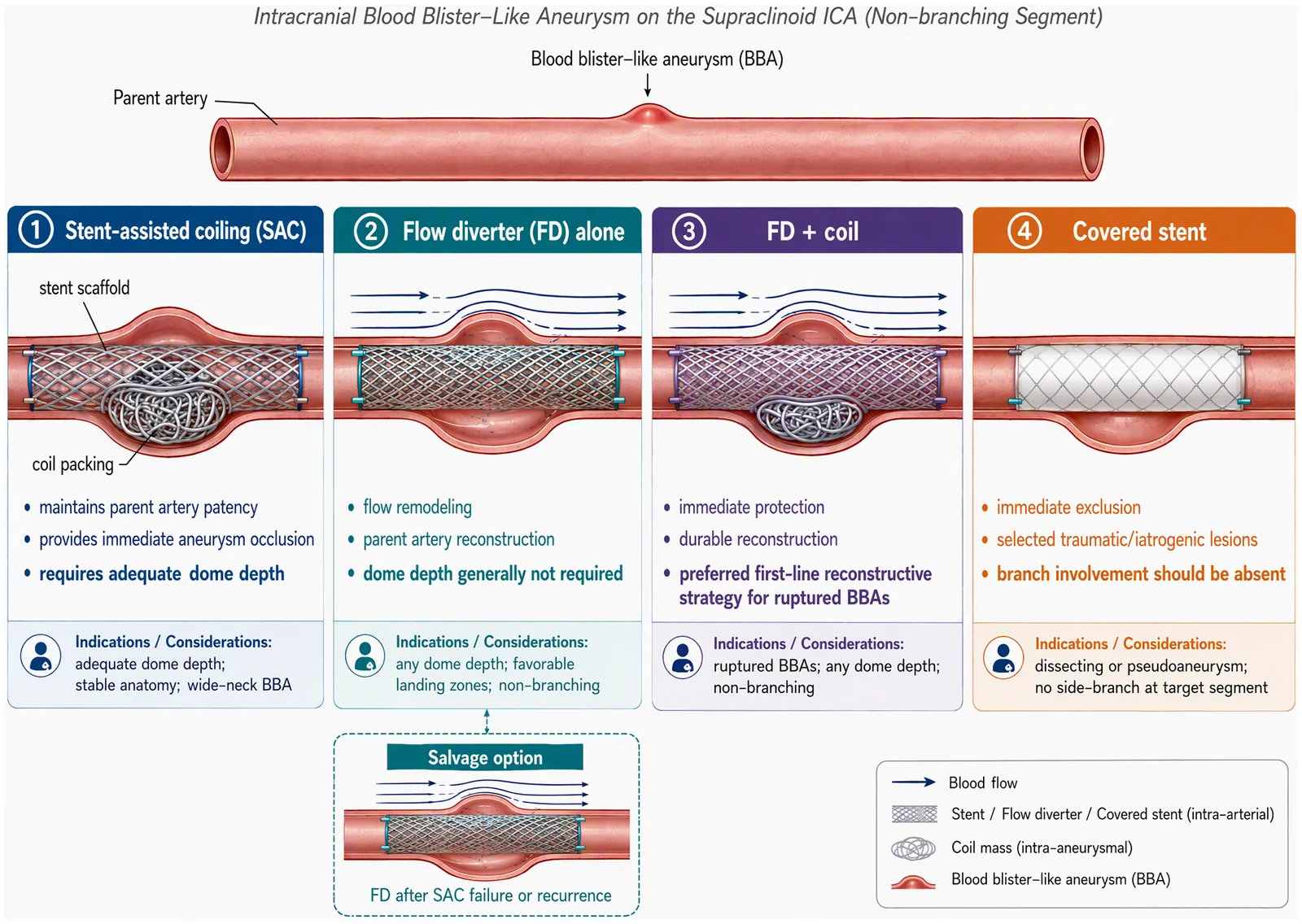
Endovascular treatment modalities for BBAs. **(A)** Stent-assisted coiling (SAC): a stent scaffold across the aneurysm neck permits dense coil packing within the sac; requires adequate aneurysm dome depth; associated with recurrence in 20–30%. **(B)** Flow-diverter (FD): a dense mesh across the neck reduces intra-aneurysmal flow and promotes thrombosis and endothelialization; no dome-depth requirement; high occlusion rates (~88%) with delayed occlusion and thromboembolic risk. **(C)** Covered stent: a physical barrier immediately excludes the aneurysm; no dome-depth requirement; suited to traumatic/iatrogenic lesions without branch involvement, with risks of branch coverage and stiff delivery. A comparative table summarizes the mechanisms, best indications, occlusion timing, antiplatelet requirements, and key risks of each modality. DAPT, dual antiplatelet therapy; FD, flow-diverter; SAC, stent-assisted coiling.

#### 2.3.3 Complications and management of endovascular therapy

Endovascular treatment of intracranial BBAs, while minimally invasive and increasingly favored, carries significant potential complications that require careful management. Commonly reported complications include thromboembolic events, rebleeding, and in-stent stenosis. Thromboembolic complications are particularly notable, especially in procedures involving FDs or stent-assisted coiling, with reported rates ranging from 5% to over 16%, depending on aneurysm complexity and the specific intervention employed (40). These ischemic events can result in substantial morbidity and even mortality, as demonstrated in studies of large basilar trunk aneurysms, where ischemic complications accounted for the majority of neurological adverse events post-procedure (41).

The management of endovascular complications is closely linked to perioperative antiplatelet therapy. Dual antiplatelet therapy is standard for patients undergoing stent or FD placement to reduce the risk of thromboembolic events; however, it simultaneously increases the risk of hemorrhagic complications, necessitating careful balancing and individualized patient assessment (40). Platelet function testing, including platelet response assays such as VerifyNow P2Y12 and light transmission aggregometry, has been increasingly utilized to guide antiplatelet therapy in complex endovascular cases. These assays enable identification of patients with high platelet reactivity (“resistance” to clopidogrel), who may benefit from alternative antiplatelet agents (e.g., prasugrel or ticagrelor) or dose adjustments to optimize antithrombotic efficacy while minimizing bleeding risk (42). Risk stratification tools, such as the SAFETEA score, have been developed to predict procedural complication risk based on patient and aneurysm characteristics, thereby facilitating perioperative risk control and personalized treatment planning (43).

Rebleeding represents another serious concern, particularly in the periprocedural period, and can be challenging to distinguish from contrast extravasation on imaging. Advanced imaging modalities, such as dual-energy CT, are recommended to differentiate these entities and guide management decisions, including the continuation or cessation of antiplatelet therapy (44). In-stent stenosis, although less common, may compromise long-term vessel patency and necessitate additional intervention. Collectively, these considerations underscore the importance of meticulous procedural planning, vigilant intra- and post-procedural monitoring, and individualized risk mitigation strategies in the endovascular management of BBAs.

### 2.4 Treatment strategies for special types of BBAs

#### 2.4.1 Traumatic and morphologically similar lesions

Although traumatic aneurysms and iatrogenic vessel injuries are occasionally described together with BBAs because they share a similar thin-walled, broad-based, blister-like morphology on imaging, they are etiologically distinct from true (spontaneous) ICA BBAs and should not be classified as such. True ICA BBAs arise from spontaneous focal dissection of the arterial wall on a background of mural degeneration and atherosclerosis, whereas traumatic/iatrogenic lesions result from direct mechanical vessel injury. We therefore address these lesions separately here, emphasizing that their management should be guided by their specific etiology.

Traumatic blister-like aneurysms are rare but pose significant therapeutic challenges due to their fragile wall structure and high propensity for rupture. Their pathogenesis involves direct vessel injury, typically near the anterior clinoid process of the ICA. These aneurysms may develop within one to two weeks following trauma and are particularly prone to rupture around the second week post-injury, necessitating prompt intervention. High-flow bypass using a radial artery graft or saphenous vein graft combined with lesion trapping has emerged as an effective deconstructive surgical strategy, especially in cases where conventional endovascular approaches are unfeasible or carry a high risk of rebleeding (45).

For traumatic blister-like aneurysms and iatrogenic vessel perforations, covered stents represent an important reconstructive endovascular option. In particular, covered coronary stents, originally developed for coronary artery disease, have been successfully adapted for the treatment of traumatic and iatrogenic lesions. These devices provide immediate complete exclusion of the lesion while preserving parent artery patency, offering a valuable alternative to surgical trapping in selected cases (46). The use of WCS for complex vascular lesions of the ICA and vertebral artery, including BBAs, has shown high rates of immediate aneurysm occlusion with low complication rates, supporting their safety and efficacy in selected cases (47). Additionally, FD placement has proven effective for recurrent aneurysms following stent-assisted coiling, including ruptured BBAs and traumatic pseudoaneurysms, yielding favorable angiographic and clinical outcomes with minimal procedural complications (34).

#### 2.4.2 Management of tiny (≤2 mm) aneurysms

The diagnosis and management of aneurysms measuring ≤2 mm pose significant clinical challenges due to their small size and subtle radiographic features. Conventional imaging modalities, including CTA and DSA, often fail to detect these microaneurysms, particularly in the acute phase of SAH, which may delay diagnosis and treatment (48, 49). Computational fluid dynamics (CFD) analysis has recently emerged as a valuable adjunct, with the parent artery radiation sign (PARS) serving as a predictive marker for microaneurysm formation adjacent to unruptured cerebral aneurysms (50).

It should be emphasized that basilar artery perforator microaneurysms are morphologically blister-like but are not true ICA BBAs: they have a distinct natural history (many resolve spontaneously), a different location, and a different pathophysiology. The natural history of microaneurysms is variable; some may spontaneously resolve under conservative management, particularly perforator aneurysms of the basilar artery, indicating that not all cases necessitate immediate intervention (48). Nevertheless, the risk of rupture and rebleeding remains significant, and recommended treatment must be individualized. Clinical experience suggests that endovascular approaches, including flow diversion, are associated with better outcomes than surgical interventions for small and very small aneurysms, as evidenced by lower morbidity rates and improved functional status at follow-up (51). However, the technical deployment of FDs remains challenging, particularly when the parent vessel is of small caliber and the aneurysm wall is extremely fragile; importantly, aneurysm dome size alone (including tiny ≤2 mm lesions) is not the same as parent-vessel diameter, and a small dome does not necessarily preclude FD deployment provided the parent artery is of adequate diameter and favorable anatomy. Non-ICA blister-like lesions are relatively rare and can be classified according to their location (e.g., basilar, vertebral, or PICA), each carrying distinct hemodynamic and technical considerations. Therefore, the decision to employ FDs should be individualized, considering aneurysm morphology, location, parent-vessel caliber, patient-specific risk factors, and the potential for spontaneous resolution versus the catastrophic consequences of rebleeding.

### 2.5 Comprehensive considerations in the selection of treatment methods

#### 2.5.1 Individualized patient assessment

Individualized assessment of patients with intracranial BBAs is essential for determining the most appropriate treatment strategy, as factors such as age, comorbidities, and arterial anatomical variations significantly influence therapeutic decisions and outcomes. For example, a systematic review of FD monotherapy in BBAs reported a mean patient age of approximately 50 years and a predominance of female patients, suggesting that demographic characteristics may impact both disease presentation and treatment response (2). Underlying conditions such as hypertension, diabetes, or other vascular comorbidities can further complicate surgical or endovascular interventions, necessitating a tailored approach that balances procedural risks with potential benefits.

Anatomical variations, particularly the frequent localization of BBAs in the ICA, highlight the importance of detailed preoperative imaging and planning to ensure optimal device selection, deployment, and anticipation of technical challenges unique to each patient (2). Comprehensive preoperative assessments, including evaluations of cerebral blood flow reserve and collateral circulation, are critical in guiding the choice between reconstructive and deconstructive treatments and in predicting the risk of ischemic complications, especially when temporary or permanent arterial occlusion may be required.

Intraoperative neurophysiologic monitoring (IONM), while widely used in aneurysm surgery, has shown limited sensitivity for detecting ischemic events during BBA procedures, particularly in complex cases involving trapping or advanced vascular reconstruction (12). These findings are derived from a single retrospective case series with a small sample size, and the overall evidence supporting routine IONM in BBA surgery remains weak. This limitation underscores the necessity of integrating multimodal preoperative assessments rather than relying solely on intraoperative monitoring to guide intraoperative and perioperative decision-making. Ultimately, a multidisciplinary evaluation that considers patient-specific clinical and anatomical factors, as well as advanced imaging and physiological testing, is crucial for optimizing outcomes in the management of intracranial BBAs (2, 12).

#### 2.5.2 Multidisciplinary collaboration and decision-making processes

The management of intracranial BBAs is inherently complex and requires a comprehensive, multidisciplinary team approach involving neurosurgery, neurointerventional radiology, anesthesiology, and critical care specialists. This collaborative model is essential due to the unique anatomical and pathological characteristics of BBAs, which often demand rapid, individualized, and sometimes hybridized treatment strategies. Neurosurgeons and neurointerventionists work closely to assess the feasibility of microsurgical clipping, bypass, or endovascular approaches, including flow diversion, stent-assisted coiling, or combined techniques, considering aneurysm morphology, patient comorbidities, and the urgency of intervention. Anesthesiologists play a critical role in perioperative management, particularly in patients with SAH or those undergoing complex vascular reconstructions, ensuring hemodynamic stability and neuroprotection during procedures (52). Critical care teams are integral to postoperative monitoring, early detection of complications, and implementation of neuroprotective protocols. Decision-making is further enhanced by advanced imaging modalities and interdisciplinary case conferences, which facilitate consensus on optimal treatment pathways and allow for dynamic adjustment of strategies in response to intraoperative findings or evolving clinical scenarios (53).

#### 2.5.3 Application of the stepwise two-pathway treatment algorithm

To translate the above considerations into practical guidance, we propose a stepwise, two-pathway treatment algorithm (Figure 2) that separates the endovascular and surgical pathways and proceeds through sequential decisions within each. This framework is intended to address the clinical challenge that no single modality is optimal for all BBAs, and to provide clinicians with an explicit, reproducible route to individualized decision-making.

In the endovascular pathway, the first decision point concerns whether the aneurysm neck is definable and reconstructable. For lesions with a definable or wide neck and adequate dome depth, stent-assisted coiling (SAC) may be considered; however, because BBAs frequently lack a definable neck and recur in 20–30% of cases, flow-diversion (FD) alone or in combination with coil embolization is generally preferred as the first-line reconstructive strategy, particularly given the higher complete-occlusion rates of FD + coil (~98%) than FD alone (~87%). Covered stents are reserved for traumatic/iatrogenic lesions without branch involvement, where immediate physical exclusion is advantageous. If a BBA recurs or is incompletely occluded after initial SAC, FD placement serves as a salvage strategy for definitive reconstruction. This algorithm emphasizes that the choice among SAC, FD, and covered stents is driven not by device availability but by aneurysm morphology, dome depth, and etiology.

In the surgical pathway, the algorithm proceeds from direct clipping (for a discrete, accessible neck with a relatively intact wall) to wrapping–clipping (for fragile or irregular walls at high rupture risk), and ultimately to deconstructive trapping with bypass for lesions unsuitable for reconstruction. Critically, the decision to proceed with a deconstructive strategy must be preceded by assessment of collateral circulation (via balloon occlusion test or imaging); deconstructive trapping is only appropriate when adequate collateral flow is confirmed, and prophylactic bypass (high-flow radial artery/saphenous vein EC-IC bypass, or low-flow STA–MCA bypass) is indicated for patients with poor predicted collateralization, planned parent-artery sacrifice, or failed reconstruction. This pathway clarifies the specific indications and limitations of each surgical technique and the circumstances that warrant a prophylactic bypass.

Collectively, this algorithm underscores that BBA management should be driven by a structured, stepwise assessment of aneurysm and patient characteristics rather than a fixed default approach, and it explicitly integrates the reconstructive-versus-deconstructive framework with location-, morphology-, and collateral-based considerations. It also serves as a practical tool for multidisciplinary teams to reach consensus and to adapt the plan dynamically as intraoperative or follow-up findings evolve.

### 2.6 Latest research progress and future directions

#### 2.6.1 New materials and devices

The clinical management of intracranial BBAs has been enhanced by the development and application of next-generation endovascular devices. Among these, next-generation FDs (e.g., PED, FRED) have demonstrated high rates of aneurysm occlusion in both anterior and posterior circulations, with long-term follow-up showing progressive complete occlusion and comparable safety profiles across devices (54); these devices are particularly valuable for wide-necked and complex aneurysms not amenable to conventional coiling or clipping (55, 56). Intrasaccular flow disruptors, such as the Woven EndoBridge (WEB) device, have shown efficacy for wide-neck bifurcation aneurysms (57, 58).

It must be emphasized, however, that most of these newer technologies and materials have not yet been validated for the specific management of BBAs, which are technically demanding lesions characterized by fragile, thin walls, small vessel caliber, and the absence of a definable neck. Devices developed for saccular bifurcation aneurysms (e.g., WEB, intrasaccular disruptors) or early-stage experimental materials (e.g., bioresorbable scaffolds, shape memory polymer embolic devices) cannot currently be recommended for routine BBA treatment, and their discussion is therefore intentionally brief. Ongoing preclinical and early clinical evaluation is required before any of these innovations can be translated to this challenging lesion type (55, 56).

#### 2.6.2 Optimization of treatment strategies and evidence-based medicine

Large-sample, multicenter clinical studies have been instrumental in optimizing treatment strategies for BBAs. Systematic reviews and meta-analyses aggregating data from multiple centers have clarified the relative safety and efficacy of various interventions, including microsurgical techniques (such as EC-IC bypass with parent vessel trapping), endovascular approaches (including FDs and covered stents), and hybrid strategies. For example, a systematic review evaluating EC-IC bypass with parent vessel trapping for ICA BBAs included 13 studies encompassing 98 patients, demonstrating that this deconstructive approach can yield favorable outcomes, while also highlighting limitations in data quality and the absence of prospective, large-scale studies (14). Similarly, multicenter case series and meta-analyses of FD monotherapy and WCS deployment have shown high rates of aneurysm occlusion and favorable functional outcomes, while emphasizing the importance of careful patient selection and standardized treatment protocols (2, 4, 15, 47). Importantly, comparative meta-analyses have demonstrated that endovascular approaches are associated with higher rates of good clinical outcome and lower rates of intraoperative rupture and mortality than microsurgery in ruptured BBAs (59, 60), and that flow-diverter treatment achieves high complete-occlusion rates across multiple series (61–64).

Looking ahead, future research in BBA management is poised to harness advances in precision medicine and artificial intelligence (AI)-assisted decision-making (65–67). Precision medicine, by integrating patient-specific factors such as genetic profiles, aneurysm morphology, and comorbidities, offers the potential to tailor interventions that maximize therapeutic efficacy while minimizing procedural risks (69, 70). The increasing availability of large, multicenter datasets will further enable the development and validation of AI-based predictive models to guide individualized treatment strategies. Advanced imaging modalities, including magnetic resonance CE-VWI, are also improving diagnostic accuracy and informing the selection of optimal therapeutic approaches (6, 68). The integration of these technologies into routine clinical practice, combined with rigorous prospective validation, will be essential for translating research findings into improved patient outcomes (71, 72).

## 3 Conclusion

Intracranial BBAs remain a formidable challenge in neurovascular medicine because of their fragile, thin-walled, broad-based morphology and high rupture risk, and no single approach is optimal for all patients. We therefore recommend a stepwise, two-pathway algorithm to guide treatment selection. In the endovascular pathway, FD + coil embolization represents a preferred first-line reconstructive strategy for ruptured BBAs given its high complete-occlusion rates and durable healing, with SAC reserved for lesions with an adequate definable dome and covered stents for traumatic/iatrogenic lesions without branch involvement. In the surgical pathway, direct clipping is suited to discrete accessible necks with relatively intact walls, wrapping-clipping to fragile or irregular walls, and deconstructive trapping with high- or low-flow bypass to lesions unsuitable for reconstruction and to patients with adequate collateral flow. Individualized, multidisciplinary decision-making—considering aneurysm morphology, location, parent-vessel caliber, collateral reserve, and institutional expertise—is essential, and prospective, multicenter studies are required to establish evidence-based guidelines for this complex cerebrovascular pathology.
